# Natural History of Idiosyncratic Drug‐Induced Liver Injury and Prognostic Models

**DOI:** 10.1111/liv.70138

**Published:** 2025-05-14

**Authors:** Harshad C. Devarbhavi, Raúl J. Andrade

**Affiliations:** ^1^ Department of Gastroenterology and Hepatology St. John's Medical College Hospital Bangalore India; ^2^ Unidad de Gestión Clínica de Enfermedades Digestivas Instituto de Investigación Biomédica de Málaga. IBIMA‐Plataforma BIONAND, Hospital Universitario Virgen de la Victoria, Universidad de Málaga, Centro de Investigación Biomédica en Red de Enfermedades Hepáticas y Digestivas, (CIBERehd) Malaga Spain

**Keywords:** chronic DILI, corticosteroids, drug‐induced acute liver failure, prognostic factors

## Abstract

Background and Aims: Drug‐induced liver injury (DILI) remains a leading cause of acute liver failure worldwide. Drugs such as isoniazid, alone or in combination with other anti‐tuberculosis drugs, as well as a growing number of herbal and complementary medicines, have been implicated in most cases of acute liver failure in registry studies. Methods: This review summarizes current knowdledge on the acute and chronic outcomes in patients with idiosyncratic DILI and discusses several of the existing prognostic models. Results and Conclusions: The reasons why some individuals progress from DILI to end‐stage liver disease are still largely unknown. However, collaborative efforts over the past few decades have provided figures on the relative incidence of drug‐induced acute liver failure and allowed the development of prognostic models to predict this worse outcome at the onset of the event. The outcome of chronic DILI is less well characterised due to the lack of sufficient follow‐up in cohort studies, but several phenotypes of DILI can progress to chronicity, and specific drugs such as nitrofurantoin or amiodarone are classic examples of agents leading to chronic forms of DILI. Therapy for drug‐induced acute liver failure and chronic DILI is mainly supportive, although some randomised clinical trials have shown beneficial effects of N‐acetylcysteine and corticosteroids.

**Summary**

- Drug‐induced acute liver failure is a devastating clinical consequence of hepatotoxicity, which is more frequent with some classes of drugs and herbs and is not yet fully preventable.
- Several clinical and biochemical scores can help identify patients with a poorer prognosis at the onset of DILI, allowing prompt referral to transplant centres.
- Chronic DILI manifests itself in a variety of phenotypes and, although rare, can lead to severe and irreversible liver injury, particularly with long‐term exposure to some drugs.
- The treatment for drug‐induced acute liver failure and chronic DILI is not well established due to a lack of properly designed and conducted clinical trials.

## 1 Introduction

Idiosyncratic drug‐induced liver injury (DILI) remains an elusive liver disease in terms of its prediction, diagnosis and prognosis, challenging the pharmaceutical industry, regulatory authorities and clinicians. Over the past two decades, largely due to collaborative national and international efforts, progress has been made in characterising the most common phenotypes of DILI, both biochemically and histologically, the causative drugs and the identification of genetic variations that predispose individuals to DILI [1]. However, data on the natural history of DILI patients have been less consistent due to limited follow‐up in many cases. A fraction of DILI cases progress to acute liver failure (DIALF). Early indicators of DIALF are altered markers of liver function (bilirubin and INR), but other predictive factors (pattern of liver damage, female sex, creatinine, ascites, hepatic encephalopathy) have been identified in large cohorts of DILI patients [2, 3]. Similarly, early predictors of chronicity in DILI have emerged in follow‐up studies. In this article, we review the causative agents, predictive factors, clinical presentation and management of the DILI more severe outcomes, DIALF and chronicity.

### 1.1 Epidemiology of DIALF and Causative Drugs

Drug‐induced liver injury is an uncommon yet significant cause of DIALF. Indeed, DILI is a major reason for drug withdrawal, with 15 of the 47 (32%) withdrawals between 1975 and 2007 [4]. Telithromycin, troglitazone, bromfenac, and nefazodone are a few significant examples of recent DIALF‐related withdrawals. The incidence rate of drug‐induced ALF is uncertain but is estimated to be very low at 1.61 [95% CI, 1.06–2.35] events per 1 000 000 person‐years [5]. However, the incidence is also affected by the background rates of infections and diseases for drugs (with the potential for hepatotoxicity) are often prescribed. A wide group or classes are listed as causing DIALF [6, 7], but only a few agents are disproportionately responsible for causing DIALF globally and include antimicrobials, antiepileptic drugs, herbal and dietary supplements (HDS), NSAIDs, and others. The causes of DIALF differ geographically, with acetaminophen (paracetamol) toxicity, either intentional or accidental, being the major cause in the West, followed by idiosyncratic drug‐induced liver injury worldwide.

Of the approximately 1000 agents thought to cause DILI [8], only about 10%–15% of them especially progress to develop DIALF [2, 6, 9]. The different agents involved are partially listed in Table 1. In addition, 10%–15% of patients with ALF are due to DIALF [2, 10, 11]. The ALFSG data identified 97 of 277 different compounds in causing DILI [9], while an Indian registry associated only 18 different agents with DIALF. Herbal and dietary supplements or complimentary medicines are increasingly linked to DILI as well as DIALF, many of whom progress to receive liver transplantation (Table 2) [2, 9, 10, 11, 12].

**TABLE 1:** Drugs associated with acute liver failure. Data collected and combined from references [3, 6, 7, 9, 10, 13].

Antibacterials	Antivirals	Antifungals	Antiseizure medications	NSAID	Statins	HDS	Misc
*Antituberculosis agents* Isoniazid monotherapy; combination of isoniazid + rifampicin + pyrazinamide *Sulfonamide* TMP‐SMX Dapsone *Macrolides* Azithromycin, Clarithromycin Roxithromycin, Telithromycin (withdrawn) *ß‐Lactams* Amoxicillin–clauvulinic acid, Amoxicillin, Flucloxacillin *Cephalosporins* Cefepime, cefazolin, cefuroxime axetile *Others* Nitrofurantoin Minocycline	*NNRTI* Nevirapine, efavirenz *NRTI* Abacavir, zidovudine, stavudine, didanosine *PI* Darunavir ritonavir, atanazavir tipranavir *CCR5 antagonist* Maraviroc	*Triazole* Itraconazole, fluconazole *Allylamine* Terbinafine *Imidazole* Ketoconazole	*First generation agents* Phenytoin sodium Carbamazepine Valproate Phenobarbitone *Second generation agents* Lamotrigine Oxcarbazepine Levetiracetam	Diclofenac Etodolac Ibuprofen Bromfenac	Atorvastatin Cervistatin Simvastatin Rosuvastatin	Herbal agents unspecified Weight loss supplements Muscle building supplements Usnic acid Garcinia cambogia Green tea extract Hydroxycut Oxylite pro	Methyldopa Chemotherapeutic agents (multiple) Leflunomide Venlafaxine Nefazodone

Abbreviations: TMP‐SMX, trimethoprim‐sulfamethoxazole; NNRTI, non‐nucleoside reverse transcriptase inhibitor; NRTI, nucleoside reverse transcriptase inhibitor; PI, protease inhibitors; CCR5,C‐C chemokine receptor type 5; NSAID, non‐steroidal anti‐inflammatory agents; Misc, miscellaneous.

**TABLE 2:** Summary of select characteristics of drug‐induced acute liver failure (DI‐ALF) studies across countries and registries (with > 100 DIALF cases).

Country	USA	USA	India	India	USA	Sweden
Author/Journal	Reuben 2010 hepatology 2010;52:2065–2076	Ghabril 2022 Liver Transpl 2022;28:169–179.	Devarbhavi liver Int. 2018;38:1322–1329	Devarbhavi J clin exp hepatol. 2021;11:288–298	Rao Am J gastroenterol 2022;117:617–626.	Bjornsson 2005 scand J gastroenterol 2005; 40: 1095–101
Numbers of DIALF patients	133 11% of 1198 ALF subjects	584 26% of 2214 waitlisted ALF patients	128 14% of 905 DILI patients	124 10% of 1288 DILI patients	277 12% of 2332 ALF patients	103 2.2% of 4687 cases
Cohort	Multicenter among ALF cohort	Multicenter among ALF cohort	Single center among DILI cohort	Multicenter among DILI cohort	Multicenter among ALF cohort	Swedish adverse drug reactions advisory committee (SADRAC)
Duration of study	1998–2007	1995–2020	1997–2017	2013–2018	1998–2018	1966–2002
Year	2010	2022	2018	2021	2022	2005
Females	71%	59%	53%	57%	69%	43%
Top 5 implicated drugs	ATT (19%) Non‐TB antibiotics (23%) HDS (10%) AED (8%) Anti‐fungal (5%)	ATT (16%) Non‐TB antibiotics (12%) AED (11%) HDS (10%) NSAID (5%)	ATT (72%) AED (17%) Non‐ATT antibiotics (7%) ART (1) Others	ATT (63%) CAM (12%) AED (8) Non‐TB antibiotics (6.4%) Others	ATT (15%) Non‐TB antibiotics (8%) HDS (15%) AED(6%) NSAID (4%) Anti‐fungals (4%)	Halothane (15%), paracetamol (11.6%), flucloxacillin (8.7%), sulfamethoxazole/trimethoprim (6%) NSAID (6%)
Spontaneous survival	27%	37%	34%	35%	30%	None

Abbreviations: AED, anti‐epileptic drugs; ALF, acute liver failure; ART, antiretroviral therapy; ATT, antituberculosis therapy; CAM, complementary and alternative medicines; HDS, herbal and dietary supplements.

Antimicrobials continue to remain the most common agent causing DIALF worldwide, with antituberculosis agents being the leading cause worldwide. Isoniazid (INH) used as a single agent (for tuberculosis prophylaxis) is common in the Western countries. However, combination antituberculosis drugs for tuberculosis disease, which consist of 3 hepatotoxic drugs (isoniazid, rifampicin and pyrazinamide) are common in India, China, and other developing countries. Isoniazid, as a single agent or in combination, accounts for 15%–19% of DIALF in the United States [6, 9, 11], compared to 63%–72% in India [3, 13] (see Table 2). The fact that INH and other antituberculosis agents continue to contribute to DIALF Cases 4–5 decades since their introduction serves as a reminder of the urgent need to monitor patients for early identification of liver injury and the necessity for novel, safer tuberculosis medications with little or no risk of hepatotoxicity. It's interesting to note that amoxicillin‐clavulanic acid, the most prevalent cause of DILI in the US, Spain, and other Western nations, seldom results in ALF [7, 11], underscoring a drug's innate propensity to cause severe liver injury.

Among anti‐seizure medication, the top three causes are first‐generation medicines including phenytoin, carbamazepine, and valproate [9, 13], while second‐generation AEDs are rarely associated with DILI and ALF. It is important to recall that the earlier generation AEDs are used for non‐seizure indications such as valproate as a mood stabiliser and carbamazepine for trigeminal neuralgia [14, 15].

Importantly, DILI due to HDS has increased worldwide and ALF owing to HDS has increased eight‐fold from 2.9% to 24.1% of all idiosyncratic DIALF patients from 1995 to 2020 [11]. Currently, HDS constitutes the second most common cause of both DILI and DIALF in the USA [11].

Two further uncommon causes of DIALF are sinusoidal obstruction syndrome (SOS) and lactic acidosis steatohepatitis (LASH). SOS may develop after a high dose of chemotherapy such as thioguanine, busulfan, cyclophosphamide, melphalan, or oxaliplatin or chemoradiation prior to haematopoietic cell transplantation [16]. The presentation is generally acute with abdominal pain, jaundice, hepatomegaly, and ascites. Among herbs, pyrrolizidine alkaloids present in Tusanqi (Gynura segetum) are a well‐known example of SOS [17]. LASH has been observed with linezolid, although it was typically associated with nucleoside analogues fialuridine, didanosine, stavudine, and zidovudine, which have either been withdrawn or replaced with better and safer drugs.

### 1.2 Phenotype Leading to Acute Liver Failure

Cholestatic injury (as defined by the ‘R’ ratio) has a generally better prognosis than hepatocellular injury, although persistent liver test abnormalities may be more common in cholestatic injury [18]. The hepatocellular liver injury pattern is more common in severe disease and DIALF, and accounts for ~90% of cases of ALF [19]. Unlike acetaminophen toxicity, which is associated with hyperacute ALF, idiosyncratic DIALF develops more slowly over days and weeks into a subacute hepatic failure form during which time there is marked hyperbilirubinemia and modest elevation of aminotransferase, with encephalopathy developing as a terminal event, as exemplified by antimicrobials, particularly antituberculosis hepatotoxicity [20]. Only in 10% of cases of DIALF does a cholestatic pattern show from disease onset, while typically the hepatocellular injury pattern develops into a cholestatic pattern at the time of transplantation [19].

Some patients with DIALF develop features of hypersensitivity including eosinophilia, skin rashes, fever, and lymphadenopathy as part of the drug reaction eosinophilia and systemic symptoms (DRESS) syndrome [21]. These are commonly associated with anti‐seizure medications such as phenytoin, carbamazepine, and lamotrigine [14, 15]. Because of short latency and early treatment with corticosteroids, the liver injury may be less severe with mortality < 10% [14] but may be higher in African‐Americans [15]. Mortality is contributed to by liver and other non‐cutaneous system involvement. Rarely, the liver may be involved in another severe cutaneous adverse reaction such as Stevens‐Johnson syndrome/Toxic Epidermal Necrolysis (SJS/TEN) with a higher fatality from significant severe cutaneous involvement and consequences [22].

### 1.3 Prognostic Models to Predict Serious Short‐Term Outcomes

Over the last 1–2 decades, there has been a gradual decrease in the number of cases of DIALF, along with improved outcomes, which has been attributed to advances in critical care and management of complications. While there are prognostic models for ALF such as King's College Hospital model, Model for End‐Stage Liver Disease (MELD) score, and ALFSG models, a few DILI‐specific prognostic models [23, 24, 25, 26, 27] are presented in Table 3 and briefly discussed below.

**TABLE 3:** Prognostic model studies in drug‐induced liver injury.

Models	Variables in model	DILI cases	Type of study	Outcome	Sensitivity	Specificity	Comments^a^
Hy's Law^b^	TB> 2 ULN, ALT> 3ULN, and ALP< 2 ULN	318	Prospective^b^	Mortality	90	44	Calculated at disease onset^a^
MELD score	10 × [(0.957 × ln(Creatinine)) + (0.378 × ln(Bilirubin)) + (1.12 × ln(INR))] + 6.43	157	Retrospective	ALF; 30days death/LT	88	72	Calculated at disease onset^a^
DrILTox ALF Score^c^	−0.00691292 × platelet count (per 10^9^/L) + 0.19091500 × TB (per 1.0 mg/dL)	15 353	Retrospective		91	76	Calculation at DILI diagnosis
nHy's Law	TB > 2ULN, nR ≥ 5, nR = ALT or aspartate ami‐notransferase (AST), whichever the highest/ULN ÷ ALP/ULN	771	Prospective	ALF	63	90	Calculated at disease onset^a^
Prognostic algo‐rithm by Robles et al.	AST > 17.3 × ULN and TB> 6.6 × ULN; or AST≤ 17.3 × ULNplusAST/ALT ratio > 1.5	771	Prospective	ALF	82	80	Calculated at disease onset^a^
Prognostic algo‐rithm by Ghabril et al.	CCI × 25.53383 + [100 + 100 × (1.5‐albu‐min)/(4.5–1.5)] × 2.7669/3.3558 + 100 × (MELD score‐6) × 0.0987/3.3558	553	Retrospective	6 month mortality/LT			Calculation at disease onset (first available labs for MELD closest to onset)
DMP score	1.913 × INR + 0.060 × TB (mg/dL) + 0.439 × AST/ALT—1.579 × ALB (g/dL)—0.006 × PLT (109/L) + 9.662	2008	Retrospective	6 month mortality/LT	88	91	Calculated at disease onset^a^

^a^ Using the first available laboratory data.

^b^ Based on MercedesRobles‐Diaz Gastroenterology 2014;147:109–118.

^c^ Drug‐induced liver toxicity ALF score.

Jaundice in the setting of hepatocellular injury predicts a severe illness and outcome. This observation, attributed to Hyman Zimmerman and that bears his name as Hy's law, is associated with the risk of death or liver transplantation in > 10% of cases [28]. This has been validated across various registries and countries, with mortality or liver transplantation of > 10% [2, 3, 29]. Hy's law has been defined as (bilirubin > 2 mg/dL) as a marker of liver dysfunction and aminotransaminases elevated (> 3 X ULN) as a marker of liver cell injury in the absence of cholestasis (alkaline phosphatase [ALP] to < 2 ULN) [28]. Hy's law is used by the FDA to predict the risk of serious hepatotoxicity of drugs in drug development, and post marketing. In a population cohort, Hy's law criteria at DILI diagnosis had high specificity (0.92) and negative predictive value (0.99), but low sensitivity (0.68) and positive predictive value (0.02) for incident ALF [25].

In an effort to increase the accuracy of Hy's law for DIALF prediction, investigators from the Spanish DILI registry discovered that AST > 17.3 ULN, TBL > 6.6 ULN, and AST:ALT > 1.5 were the most appropriate cut‐off levels, identifying patients who developed ALF with 82% specificity and 80% sensitivity [23]. Patients in this cohort had advanced liver disease, with 66% exhibiting jaundice and 52% requiring hospitalisation.

Patients with DILI often recover from the hepatocellular injury but die as a result of accompanying comorbidities. In the DILIN study on the cause of death, DILI played a primary role in 64% of patients, whereas a contributory role and no role were discovered in 14% and 21%, respectively [17].The authors found that the nR Hy's Law was a better measure of the risk of death than the original Hy's Law [19]. The Spanish registry and the US DILIN registry both suggest nR‐value based definitions outperform the ALP no higher than twice normal criterion for identifying Hy's Law cases. Whether the same is true in clinical trials is still unclear.

Model for End‐Stage Liver Disease (MELD) although originally developed for candidacy for liver transplantation and TIPS, tends to perform well in assessing mortality in DILI, with fairly good sensitivity (0.80) but modest specificity (0.72) [24].

The role of comorbidities on outcome in DILI patients was included in a model to predict 6‐month mortality. This model consisted of Charlson Comorbidity Index scores higher than 2, Model for End‐Stage Liver Disease (MELD), and serum albumin to predict 6‐month mortality. A model based on these 3 variables identified patients who died within 6 months, with c‐statistic values of 0.89 (95% CI, 0.86–0.94) in the discovery cohort and 0.91 (95% CI, 0.83–0.99) in the validation cohort. Despite the publication of several predictors of risk of ALF, there have not been any changes to the current regulatory guidelines pertaining to drug development [30].

However, the annual rate of waitlisting for newer agents has decreased over the last 2 decades, attributed to drug development guidance as well as increased attention to risks of hepatotoxicity by regulatory and postmarketing oversight [11] Furthermore, the MELD score, which incorporates two variables involving liver function (INR and bilirubin) [31] and has been shown to perform well in a variety of liver diseases, including DILI and ALF, appears to be the most universally applicable model for DILI and ALF.

Another DILI mortality predictive (DMP) model developed by Chinese investigators performed well and was driven by the need to assess the risks of traditional medicine induced liver injury in that population [27].

### 1.4 Histology Prognostic Features

Liver biopsy is generally not recommended for diagnostic purposes in DILI except in rare cases such as for confirmation or strengthening of a diagnosis of drug‐induced autoimmune‐like hepatitis [32]. In other instances, it may be used when liver biochemistry does not improve despite discontinuation of an implicated drug. The presence of extensive hepatocyte, ductular reaction, microvesicular steatosis, cholangiolar cholestasis, fibrosis, and portal venopathy was associated with ALF, death, or liver transplantation [33]. Conversely, the presence of granulomas and eosinophils is associated with a good outcome, while liver necrosis portends a poor outcome [12, 32]. In one study that analysed liver specimens of 74 patients with acute liver failure and severe subacute liver impairment, which included at least a third of patients with DILI, the authors found the percentage of hepatocyte loss, number of proliferating hepatocytes, and number of hepatic progenitor cells to be associated with survival or death/transplantation. Surviving patients had significantly less hepatocyte loss, less hepatocyte progenitor cell activation, and more mature hepatocyte proliferative activity compared with those who either died or were transplanted [34].

In patients with prolonged cholestasis, liver biopsy may be required to determine the presence of interlobular bile ducts. Ductopenia confirmed by CK7 (cytokeratin 7) suggests a lack of bile ducts or vanishing bile duct syndrome (VBDS).

Although a large proportion of patients with VBDS are treated with corticosteroids and ursodiol, there is little evidence of their effectiveness [35].

### 1.5 Acute on Chronic Liver Failure Caused by Idiosyncratic Hepatotoxicity

The rising prevalence of obesity, diabetes mellitus, and steatotic liver disease has focused attention on the risks and outcomes of DILI in such patients [36]. There are two issues that must be addressed here. The first question is whether underlying liver illness is a risk factor for DILI, and the second is whether people with underlying liver disease and cirrhosis have a worse result.

The increased risk of hepatotoxicity with anti‐TB medicines in patients with underlying hepatitis B and C was the early indication of underlying liver disease as a risk factor for DILI. The risks are greater in patients with HBeAg positive status and higher HBV DNA. Nonetheless, meta‐analyses have revealed a 2–3 fold increased risk of hepatotoxicity with anti‐TB medicines [37]. With regard to MASLD, two studies, one each from Italy and the USA and one letter to the editor from Korea have demonstrated a 4‐fold risk of DILI in patients with underlying steatotic liver disease [38, 39, 40, 41]. However, real‐life experience appears to be at odds with the above study results. For example, disease‐specific alterations in the functioning of cytochrome P 450 enzymes, uptake, and efflux transporters occur, leading to alterations in absorption, distribution, metabolism, and excretion of many drugs. These result in increased drug exposure within hepatocytes and systemically, thereby raising the potential for adverse drug reactions [40].

DILI can act as a triggering factor in patients with underlying liver disease or cirrhosis, resulting in a worse outcome. The poor outcome is a reflection of inadequate functional reserves that limit the liver's ability to recover from the injury [42]. Labelled as ACLF, this entity results in increased mortality and the need for transplantation. Drugs constitute 2%–10.5% of causes of ACLF [43]. In the Drug‐Induced Liver Injury Network registry, 16% of 89 patients with underlying CLD who had surrogate markers of NAFLD died compared to 5.2% of 800 patients without underlying CLD (*p* < 0.001) [44], while in the Spanish DILI registry, 53 (7.5%) patients with underlying CLD died compared to 14 of 790 (1.8%) patients without underlying CLD (*p* = 0.02) [45]. Most of the deaths were caused by DILI due to antimicrobials. A summary of published reports on drug‐induced ACLF [43, 46, 47, 48, 49, 50, 51, 52, 53, 54, 55] are shown in Table 4. While antimicrobials are the most common triggering factors, there is a greater incidence of herbal agents causing ACLF worldwide. There was a trend for increased waitlist mortality [11] and decreased transplantation in patients with ACLF compared to DIALF [19].

**TABLE 4:** Characteristics and clinical outcomes of patients evaluating drug‐induced acute‐on‐chronic liver failure.

Center type and patient numbers	Proportion of DILI‐ACLF	DILI causing agents	Most common aetiology of CLD	Mortality	Comments	Author/place/year
Multi center, *N* = 200; 41 with CLD	4/41 (9.7%)	Complementary and alternative medicine^a^	HBV and NAFLD	4.8%	Patients with CLD who had poor outcomes tended to have higher AST, ALT, and total bilirubin	Chirapongsathorn et al. Thailand, 2023 [46]
Multi center, *N* = 43	22/43 (51.2%)	*Tinospora cordifolia* herb	NAFLD	Four patients (9.3%) with ACLF died and one with acute decompensation received liver transplant	Giloy is associated with acute hepatitis with autoimmune features and can unmask AIH in people with silent AIH‐related CLD	Kulkarni et al. India, 2022 [14]
Single center, *N* = 27, SAH + CAM vs. SAH	All 27 included had ACLF due to SAH + biopsy proven DILI	Complementary and traditional herbal drugs and supplements	Alcohol	82% at 6 months	Patients with SAH on CAM therapy had very poor transplant free survival and specific features on liver biopsy	Philips et al. India, 2019
Single center, *N* = 343	3/343 (0.9%)	Not detailed^a^	HBV	ACLF due to hepatic insult including DILI –73%	Hepatic insults leading to ACLF had significantly higher 28 days mortality than non‐hepatic insults	Maipang et al. Thailand, 2019 [19]
Single center, *N* = 145	33/145 (22.8%)	*Polygonum multiflorum* Thunb.	ALD	Higher mortality in those with CLD vs. those without (9% vs. 0.9%)	Concurrence of pre‐existing CLD an independent risk factor for both of chronicity and mortality with *Polygonum* herb‐liver injury	Jing et al. China, 2019 [13]
Multinational, multicenter, *N* = 3132	329/3132 (10.5%)	CAM (72%)^a^ AntiTB drugs (27%)	Alcohol	DILI: 47%, No‐DILI: 39%, at 90 days	Arterial lactate and total bilirubin independent predictors of death	Devarbhavi et al. Asia, 2019 [12]
Single center, 1132/1666 cirrhotics used CAM	35.7% (30/84)	CAM^a^	NAFLD	53% died, median survival 194 days, median follow up 173 days	CLIF‐C‐OF > 10 and HE at baseline predicted 1 month mortality, grade of ACLF at admission predicted 12 month mortality.	Philips et al. India, 2019 [20]
Single center, *N* = 300	7/300 (2.3%)	Not detailed^a^	HBV	90 days, 50%	Use of drugs and herbals did not predict development of ACLF	Li et al. China, 2016 [17]
Single center, *N* = 213	11/132 (5.2%)	Anti‐TB drugs	Alcohol	Overall: 43%, Anti‐TB: 55%	Anti‐TB‐DILI did not independently predict mortality	Shalimar et al. India, 2016
Multicenter, 1049 ACLF, *N* = 381 (DILI)	17/381 (4.5%)	Anti‐TB drugs, Antiepileptic drugs	Alcohol	Overall: 39%, DILI: 34%	Outcomes with ACLF due to DILI was not different from other acute aetiologies	Shalimar et al. India, 2016 [18]
Single center, *N* = 322	10/322 (2.5%)	Not detailed^a^	HBV	Hepatic insult including DILI: 59%	Patients with extrahepatic organ failures ACLF had significantly higher 3 month and 12 month mortality	Shi et al. China, 2015 [16]

Abbreviations: ACLF, acute‐on‐chronic liver failure; AIH, autoimmune hepatitis; ALD, alcohol‐related liver disease; ALT, alanine aminotransferase; AST, aspartate aminotransferase; AnitTB, anti tuberculosis; ATT, Anti‐Tuberculosis Treatment; AYUSH, Ayurveda; Yoga & Naturopathy, Unani, Siddha, Sowa Rigpa and Homoeopathy; CAM, complementary and alternative medicine; CLD, chronic liver disease; CLIF‐C, chronic liver failure consortium; DILI, drug‐induced liver injury; HBV, hepatitis B virus; HCV, hepatitis C virus; HE, hepatic encephalopathy; NAFLD, non‐alcoholic fatty liver disease; SAH, severe alcohol‐associated hepatitis; TB, Tuberculosis.

^a^ Ingredients not listed.

## 2 Chronic DILI Outcome

The long‐term outcome of people who have experienced a DILI event is poorly reported due to a lack of follow‐up in many studies. Various studies using different designs have addressed the issue of chronic DILI outcome. A retrospective study from the UK of liver biopsies performed in patients with suspected DILI showed that 13 out of 33 had evidence of persistent liver injury (biochemical or imaging abnormalities), with a minimum follow‐up of 1 year after the onset of DILI, and 3 of the 5 repeat liver biopsies performed showed significant changes. Factors that predicted chronic liver disease in this study were fibrosis and continued exposure to the drug [56]. Another study that analysed 685 patients with jaundice who survived the DILI episode and were linked to the Swedish Hospital Discharge and Cause of Death Registries found that 23 had been hospitalised for liver disease, 8 patients had developed cirrhosis at a mean follow‐up of 10 years, and 5 of these had ‘cryptogenic’ cirrhosis, suggesting that DILI may play a role. As in the UK study, patients with liver‐related morbidity/mortality had a longer duration of therapy before the DILI event [57]. However, the retrospective design of these studies is an important limitation in establishing a causal relationship between DILI and severe chronic liver disease.

Although reversal of liver damage is the usual outcome in DILI, the time to resolution can be variable, and in some cases of DILI, liver biochemistry abnormalities take a long time to normalise. Cholestatic DILI tends to resolve slower than hepatocellular DILI over a 12‐month follow‐up in a study [19]. Altogether, these particularities have made it difficult to define chronicity in DILI. For example, the incidence of chronic DILI in prospective studies varies between 8% and 21% depending on the time to resolution used as a cut‐off point, with most studies relying on abnormal laboratory tests rather than liver histology [19]. While at the international consensus meeting on DILI held in 1989, the authors considered chronicity to be the evidence of persistence 3 months after DILI onset [58], the AASLD practice guidance on DILI defines chronic DILI as persistent elevations in serum liver biochemistry or the presence of radiological or histological evidence of ongoing injury 6–12 months after the onset of DILI [59]. The only prospective 3‐year study published to date, from the Spanish registry, found that a significant proportion of patients continued to normalise transaminases beyond 6 months after the onset of DILI, with no differences regardless of the pattern of liver damage, suggesting that a 1‐year cut‐off point for persistent laboratory abnormalities may be a more realistic definition of chronicity in DILI [60], which has been endorsed by the EASL clinical practice guidelines [61].

Factors of susceptibility to chronic DILI outcome include older age, African‐American race, cholestatic damage [18], greater severity at presentation, and dyslipidemia [60]. Persistence of abnormal liver biochemistry has proved to be an early predictor of chronicity. Thus, bilirubin and alkaline phosphatase levels above normal values at 2 months after DILI onset showed an AUROC of 0.91 and 0.89, respectively, in predicting chronic outcome [60].

Chronicity in DILI does not seem to be, in general, a drug‐specific effect but rather associated with host‐dependent factors. Hence, causative drugs implicated in DILI chronicity vary across studies and include statins, bentazepam, fenofibrate, oral contraceptives, isoniazid, sulphonamides and trimethoprim, nitrofurantoin, ebrotidine, amoxicillin‐clavulanate, methotrexate, and terbinafine, among others [60, 61, 62, 63] (Table 5). Exceptions are some drugs, such as nitrofurantoin, methotrexate, and bentazepam, which have been reported to cause mild liver damage, allowing continued drug administration to lead to chronic damage, fibrosis, and even cirrhosis [62, 64, 65]. Some other examples are drugs that cause hepatic steatosis or steatohepatitis either by promoting weight gain and insulin resistance, such as new psychotropic agents, or causing mitochondrial dysfunction, defective lipophagy, and phospholipidosis (i.e., amiodarone) [66] In fact, amiodarone‐induced liver injury can occasionally mimic alcoholic cirrhosis [67].

**TABLE 5:** Drugs and HDS most commonly associated with chronic liver injury. Data collected and combined from references [56, 57, 60, 62, 63, 64, 65, 66, 67, 68, 70, 71, 72, 75, 76, 77, 78, 85, 86].

Antinfectives	Antiseizure medications	Anti‐rheumatic agents	Lipid Lowering agents	HDS	Misc
*Antituberculosis agents* Isoniazid Pyrazinamide^a^ *Sulfonamides* TMP‐SMX^a^ *Macrolides* Azithromycin^a^ Clarithromycin *ß‐Lactams* Amoxicillin–clauvulinic acid^a^ Amoxicillin^a^ Flucloxacillin^a^ *Others* Nitrofurantoin^b^ Minocycline^b^ *Antifungals* Itraconazole, Terbinafine	*First generation agents* Phenytoin sodium Carbamazepine Valproate Phenobarbital *Second generation agents* Lamotrigine^b^	*NSAIDs* Diclofenac Ibuprofen^a^ Celecoxib, Phenylbutazone *Others* Allopurinol^a^ Azathioprine Methotrexate	*Statins* Atorvastatin^b^, ^c^ Fluvastatin^b^ Simvastatin^b^ Rosuvastatin *Fibrates* Fenofibrate	*Traditional Chinese Medicine* *Polygonum multiflorum* Thunb *Corydalis yanhusuo* W. T. Wang *Psoralea corylifolia* L. *Bupleurum chinense* DC. *Dictamnus dasycarpus* Turcz *Terminalia chebula* Retz Artemisinin^a^	*Anaesthetic* Ketamine^c^ *Antivirals* Nevirapine^a^ *Benzodiazepines* Bentazepam *Cardiovascular agents* Amiodarone Hydrochlorothiazide, Enalapril Methyldopa^b^ *Chemotherapeutic agents (multiple)* Temozolomide Oxaliplatin Abemaciclic *ICIs* Nivolumab^c^ Pembrolizumab^c^ *Hormones* Oestrogens *H2 receptor antagonist* Ebrotidine^d^

Abbreviations: TMP‐SMX, trimethoprim‐sulfamethoxazole; NSAID, non‐steroidal anti‐inflammatory agent; Misc, miscellaneous.

^a^ Associated with vanishing bile duct syndrome (VBDS).

^b^ Associated with drug‐induced autoimmune‐like hepatitis (DI‐ALH).

^c^ Associated with secondary sclerosing cholangitis (SSC).

^d^ Withdrawn from the market.

### 2.1 Chronic Phenotypes of DILI

Several drug‐induced histopathological liver lesions can become chronic and mimic other liver diseases. Some of these, such as vanishing bile duct syndrome (VBDS) and secondary sclerosing cholangitis (SSC), are discussed in detail in a separate article in this monograph [68]. Others are briefly discussed here.

#### 2.1.1 Drug‐Induced Autoimmune‐Like Hepatitis

A phenotype of DILI potentially associated with chronic, non‐resolving liver damage is so‐called drug‐induced autoimmune‐like hepatitis (DI‐ALH), in which the clinical, laboratory and histological features may be indistinguishable from those of idiopathic autoimmune hepatitis [69]. While there are some drugs that convincingly cause DI‐ALH (i.e., nitrofurantoin, minocycline, infliximab and statins) [70, 71], in many cases of isolated case reports of autoimmune hepatitis following drug exposure, the suspected drug may be an innocent bystander and the association may be coincidental. Nevertheless, the list of drugs and herbs thought to be associated with DI‐ALH is long and growing [72], and includes SARs‐CoV‐2 vaccines, which are the subject of a dedicated article in this monograph [73]. As there are currently no specific biomarkers for AIH or DILI, the existence of DI‐ALH is debated. One proposed feature that may differentiate the two entities is the lack of relapse in DI‐ALH after spontaneous or corticosteroid‐driven resolution, in contrast to AIH [74]. Thus, formally, DI‐ALH would not meet the criteria for chronicity and would not require long‐term follow‐up. However, a DILI prospective registry study found an increase in relapses over time in DI‐ALH patients, with the absence of peripheral eosinophilia and elevated levels of transaminases and total bilirubin at presentation being factors associated with relapse [71]. Notably, in this study, long‐term statin exposure was more prevalent among DI‐ALH patients who relapsed [71], suggesting that these drugs, which have been shown to activate lupus erythematosus, may play a role in the relapse of DI‐ALH due to their immunomodulatory properties [71]. Cases initially diagnosed as DI‐ALH that relapse shortly after withdrawal of immunosuppression may not be distinguishable from ‘classic’ AIH, but a fraction of these may represent latent AIH unmasked by the drug.

#### 2.1.2 Drug‐Induced Vascular Injury

Drug‐induced vascular injury to the liver can affect various vascular structures in the organ, from the small portal veins to the large hepatic veins, and sometimes has a chronic outcome although it is also a cause of drug‐induced acute liver failure as mentioned above. Among the various injuries to the vascular structures of the liver, sinusoidal obstruction syndrome (SOS) is one that often leads to irreversible chronic liver disease, portal hypertension and its complications, although an acute phase with transition to chronicity cannot be documented in many cases. A number of herbs containing pyrrolizidine alkaloids and several drugs, including cyclophosphamide, azathioprine, mercaptopurine and thioguanine, dacarbazine, and gemtuzumab, have been associated with SOS, but nowadays oxaliplatin‐based chemotherapy used as an adjuvant treatment in patients with advanced colorectal cancer and liver metastases is the best documented cause of chronic liver injury as a result of SOS. Sinusoidal injury associated with oxaliplatin is a silent damage that occurs without clinically apparent liver injury or significant abnormalities in liver enzymes in the majority of cases [75]. The incidence of chronic (non‐reversible) portal hypertension associated with oxaliplatin was measured by imaging in a large retrospective cohort study of 356 patients with a mean follow‐up of approximately 5 years. Using parenchymal heterogeneity on CT scans as a surrogate for vascular changes, 90% of subjects receiving oxaliplatin‐based therapy were affected, and these changes were reversed in 68% of patients within 1 year, but findings of portal hypertension persisted (and progressed) until the last follow‐up in 1.4% of patients [76].A recent study using liver organoids derived from non‐cancerous liver tissue from patients has shed light on the mechanisms of oxaliplatin hepatotoxicity and the potential for prediction, showing that in those who presented with high‐grade liver injury to the oxaliplatin regimen, there was an obvious increase in mitochondrial superoxide levels and a significant decrease in mitochondrial membrane potential, which was not observed in the organoids from patients with low‐grade liver injury [77].

A minimal fraction of patients with chronic DILI may develop cirrhosis and manifestations of portal hypertension, which are more common with certain drugs such as methotrexate and amiodarone with chronic intake [78, 79].

### 2.2 Therapy of Drug‐Induced Acute Liver Failure and Chronic DILI

In most cases of DILI, discontinuation of the implicated drug and providing supportive care is sufficient to recover from liver injury. However, in a small number of cases, jaundice and liver biochemistry may worsen over time, necessitating additional measures, and transfer to advanced centres including liver transplant centres should be considered. On the other hand, long‐term follow‐up of chronic DILI to detect liver sequelae is not well established, but twice‐yearly laboratory monitoring and annual liver elastography would be a conservative approach [79].

There are only a few agents that are considered antidotes for DILI, and only a few of these have undergone randomised controlled trials. Due to the lack of strong evidence, some of these medications are used empirically.

### 2.3 N‐Acetylcysteine (NAC)

NAC has been used both for prevention and treatment of DILI including DIALF. N‐Acetylcysteine (NAC) is used as an antidote in acetaminophen (paracetamol) toxicity. However, it has been studied in acute liver failure from other causes as well as severe non‐acetaminophen DILI, and it appears to be effective in severe non‐acetaminophen DILI. This was explored in a randomised placebo‐controlled trial for non‐acetaminophen‐induced ALF with 45 DILI patients as one of the subgroups. Patients with non‐acetaminophen‐induced ALF who received NAC had a 58% transplant‐free survival compared to 27% without NAC (*p* < 0.05) [80]. Furthermore, the benefits of intravenous NAC in improving transplant‐free survival were seen in patients with early stage non‐acetaminophen‐related acute liver failure (coma grade 1‐II) [81]. The utility of NAC should be considered in the paediatric population as well.

A majority of NAC studies have focused on ATT DILI. A 2017 randomised study from India of 80 patients with ALF, 15 of whom were due to DILI mostly ATT (12/15 = 80%), found 29/40 (72.5%) survival in the NAC group vs. 19/40 (47%) in the non‐NAC group [81].

A 2021 randomised controlled trial of intravenous NAC study from South Africa investigated the efficacy of NAC in the management of ATT DILI in 102 patients [82]. Although NAC did not reduce time to ALT < 100 U/L (considered as a surrogate marker for recovery), it did significantly reduce hospital stay (*p* = 009). It is worth noting that it is important to mention that 87% of ATT patients had concomitant HIV, suggesting that ART may have a significant effect on the pathophysiology of DILI [82].

Another 2023 randomised study reported in an abstract form used oral NAC 1200/day for 2 months in the prevention of ATT DILI. The results showed a significant reduction in the incidence of ATT DILI in the NAC group compared to those who did not get NAC (0% vs. 50%; *p* = 0.02). This effect was predominantly seen in patients with NAT2 slow acetylator status.

Overall, even though it appears that NAC has the ability to improve liver biochemistry, more studies in well‐established severe DILI patients who meet the requirement of causality assessment need to be done.

### 2.4 Corticosteroids

There is insufficient evidence regarding the beneficial effects of corticosteroids in patients with severe DILI and drug‐induced ALF. Corticosteroids are commonly administered to treat patients with immunoallergic features such as drug reaction eosinophilia and systemic symptoms (DRESS) [21], or drug‐induced autoimmune‐like hepatitis (DIALH) [74, 83] as well as hepatitis caused by immune checkpoint inhibitors, although no randomised trials have been conducted to compare corticosteroids to a placebo or another agent. In DRESS syndrome, the liver involvement is often severe and together with the skin reaction may also necessitate the use and continuation of steroids which are typically tapered over 1–3 months. Early and abrupt withdrawal of corticosteroids may result in return of both cutaneous and liver injury. In DIALH, corticosteroids may be required to accelerate the normalisation of liver biochemistry and in those whose liver injury worsens despite prompt discontinuation of the causative drug [71]. In patients with ICI injury, steroids may be recommended in those with moderate to severe injury (after discontinuation of the implicated agent) with the option of second‐line medication such as MMF although this regimen is rarely used [71]. It's use in severe DILI is not associated with worse outcome, although there is a greater rate of normalisation of liver enzymes [84].

Corticosteroids have also been tried in chronic DILI. In a Chinese randomised open‐label trial, 70 patients with predominantly hepatocellular chronic drug‐ or herb‐induced liver injury(biochemical and/or histological abnormalities 6 months after the onset of DILI) were given either a stepwise dose reduction of methylprednisolone plus glycyrrhizin or glycyrrhizin alone over 48 weeks. The steroid plus glycyrrhizin group had a higher proportion of patients with sustained biochemical response (94% vs. 71%, *p* = 0.023) and a shorter time for biochemical normalisation as well as improvements in histological activity and fibrosis [85]. The same group later conducted another randomised open‐label study in 90 patients with chronic DILI showing that a 36‐week step‐down steroid regimen was as effective as a 48‐week regimen in improving liver tests, histological activity and fibrosis. Interestingly, 41% of patients had autoimmune features indistinguishable from ‘the novo’ autoimmune hepatitis (DI‐ALH) and there were no differences in the response of this group compared with the chronic DILI group without autoimmune features [86]. These trials have enrolled the larger number of patients with chronic DILI so far, although in more than half the events were adjudicated to herbal compounds, which limits the generalisability of the results.

A systematic review on the use of corticosteroids identified 24 studies/reports: the break up was as follows–moderate and severe DILI (*n* = 8) (DI‐AIH) (*n* = 5), drug‐induced fulminant acute liver failure (*n* = 2) immune checkpoint inhibitors DILI (*n* = 9) [87]. The authors found steroids to be beneficial in moderate to severe DILI, including drug‐induced ALH and ICI hepatitis, but not drug‐induced ALF [87].

Despite the putative benefits of corticosteroids in certain subsets of DILI, placebo‐controlled trials are needed to evaluate the proper indications, dose, duration, efficacy, and side‐effects.

### 2.5 Cholestyramine

Cholestyramine is a bile acid resin that has been proposed as a treatment measure in leflunomide hepatotoxicity. Leflunomide metabolites stay in the bloodstream for a long time; the active metabolite A77 1726 can be found in circulation for up to 2 years, and oral cholestyramine 8 g tid for 11 days has been recommended due to its ability to disrupt the enterohepatic circulation of leflunomide and its metabolites, along with bile acids, with increased elimination from the gut [88]. However, liver injury can develop despite the use of cholestyramine because of the idiosyncratic nature of DILI [89]. Cholestyramine is also recommended in the treatment of pruritus as a part of drug‐induced cholestasis due to many medications.

### 2.6 Ursodeoxycholic Acid

Due to its pleiotropic hepatoprotective properties and well‐established safety, ursodeoxycholic acid (UDCA) has been used empirically in DILI, mostly in the treatment of cholestatic rather than hepatocellular DILI, to prevent chronicity. Two recent systematic reviews found UDCA improved liver biochemistry variables such as bilirubin and transaminases together while also decreasing the time to liver biochemistry normalisation [61, 90]. These effects were observed within a few weeks of therapy initiation, with treatment duration extending from 2–4 weeks to 6 months [90]. UDCA dosages varied between 10 and 15 mg/kg body weight, with greater dosages used in rare instances. In some cases of marked hyperbilirubinemia, UDCA was combined with prednisolone, while in others it was replaced by plasmapheresis sessions [91]. All societies have recommended UDCA in specific settings [59, 61, 92] particularly for DILI associated pruritus [59]. However, design limitations and a lack of randomised trials comparing DILI to placebos make it impossible to make firm recommendations on its use with certainty.

### 2.7 L‐Carnitine

A 2001 study found intravenous L‐carnitine effective in the treatment of valproate hepatotoxicity [93]. Studies that replicate L‐carnitine's effect are required but may not be forthcoming due to the increased usage of newer generation antiseizure medications with greater safety profiles, including less hepatotoxicity than earlier generation agents such as valproate.

### 2.8 Other Novel Therapeutic Agents

A number of agents have been tried in DILI and other causes of liver injury. These include bicyclol, S‐adenosylmethionine, magnesium isoglycyrrhizinate (MgIG), silymarin, plasma exchange, calmangafodipir, among others. The study subjects were heterogeneous and the primary outcomes were mostly improvements in laboratory markers such as AST and ALT, which do not accurately indicate the severity of impairment. A recent systematic review provides a more in‐depth review of these novel therapies in DILI [94].

## Conflicts of Interest

The authors declare no conflicts of interest.

## Data Availability

The authors have nothing to report.
